# Use of ROC curves in clinical and experimental studies

**DOI:** 10.1590/1677-5449.200186

**Published:** 2020-12-11

**Authors:** Tatiana Cristina Figueira Polo, Hélio Amante Miot

**Affiliations:** 1 Universidade Estadual Paulista – UNESP, Faculdade de Medicina de Botucatu, Departamento de Dermatologia e Radioterapia, Botucatu, SP, Brasil.

Decision-making in clinical practice and operationalization in research are both dependent on precise and objective definitions of phenomena or outcomes (e.g. sick vs. healthy, severe vs. mild, operable vs. inoperable). However, such classifications are not always direct or unequivocal and secondary elements may be needed for categorization. Moreover, several conditions have more than one system that can be used for diagnosis, such as *diabetes mellitus* (fasting glycemia vs. oral glucose tolerance test), critical ischemia (clinical parameters vs. percentage arterial obstruction), or depression (DSM V criteria vs. the Beck inventory), and each classification has different sensitivity and specificity.^1^^-^3

In truth, in the majority of cases the criteria used to classify outcomes are not completely predictive, leading to incorrect classification of a proportion of sick (false negatives) or healthy individuals (false positives), so it is important to compare the effectiveness of the different classification systems.

A series of statistical estimators are used to analyze the performance of classificatory models and one of the most widely used is the receiver operating characteristic (ROC) curve. This is a graphical representation of the performance of a quantitative data model plotting its sensitivity (proportion of true positives) against the proportion of false positives (1-specificity) for different test values.^4^^,^^5^ Classificatory systems based on clinical symptoms, diagnostic scales, radiological findings, assays of different substances and, primarily, choice of the optimal cutoff points to maximize the performance of diagnostic tests are the most common applications for ROC curves.^6^^-^11

Figure 1A illustrates an example of a hypothetical test with high sensitivity and high specificity for diagnostic classification. This hypothetical test (test 1) has two distribution curves for the test results of sick and healthy individuals. Point A1 is the value at which best performance is achieved, considering both false positive and false negative rates (the point of maximum entropy). In turn, point A2 offers maximum specificity, because values higher than this point will not classify any false negatives, while point A3 is the point at which greatest sensitivity is reached, since values below this point will not classify any false positives. A duplex scan of the carotid is one example of a test that has this type of performance, with findings that are highly predictive of carotid stenosis.^12^

**Figure 1:**
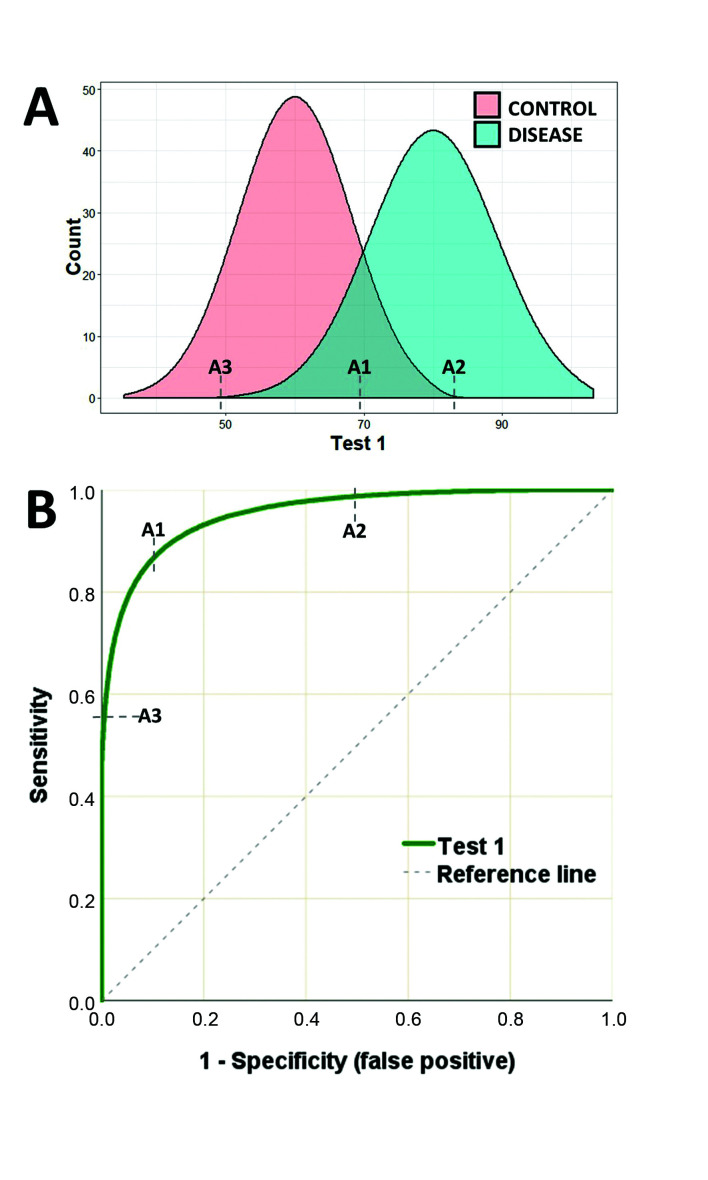
A) Illustration of the distribution curves of results of a hypothetical test to classify patients as sick or healthy. B) ROC curve for the results of test 1 plotting sensitivity against false positive rate. Point A1 is the test value (cutoff point) with greatest sensitivity and specificity (greatest proximity to the upper-left corner of the graph). Point A2 is the test value above which maximum sensitivity is achieved (zero false positive). Point A3 is the point of maximum specificity, below which there will be no false negatives.

The ROC curve for test 1 (Figure 1B) illustrates how sensitivity and specificity vary as cutoff points change, making it easy to identify points A1, A2, and A3. As a cutoff point with higher sensitivity is chosen, the diagnostic classification is unavoidably penalized by lower specificity, and vice-versa.

The closer the ROC curve approaches to the top left corner of the graph, the better the quality of the test in terms of its capacity to discriminate between groups. Moreover, the diagonal reference line on the ROC graph equates to a totally random region, where a test is incapable of classifying either healthy or sick individuals (sensitivity = specificity).

It is also possible to compare the performance of two or more classificatory models (or diagnostic tests) simultaneously using their ROC curves. Figure 2 illustrates two other tests (tests 2 and 3) for classifying sick and healthy individuals. The results curves for test 2 (Figure 2A) illustrate that there is a certain degree of superimposition of values from cases over values from controls, but show that the test performs well at low values (high sensitivity). A D-dimer assay for diagnosis of deep venous thrombosis is one example of a test with this behavior: very low values safely rule out the disease, but high values need additional confirmation (risk of false positive).^13^

**Figure 2:**
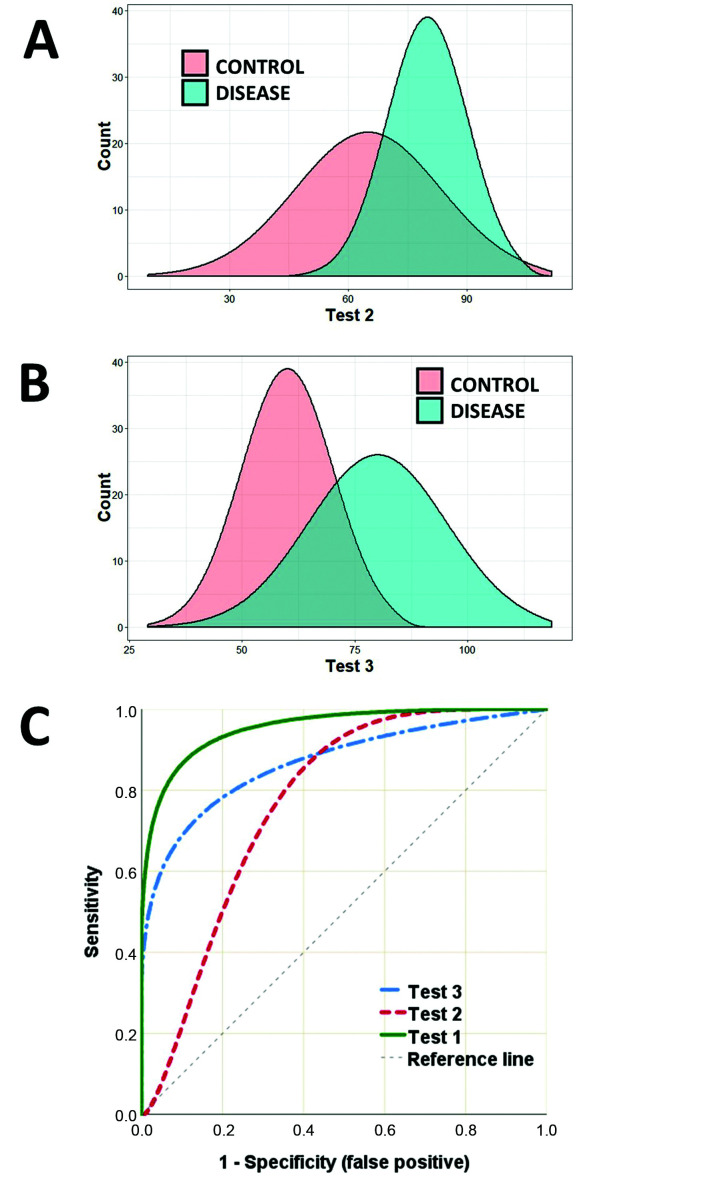
A and B) Illustration of the distribution curves for the results of tests (tests 2 and 3) to classify patients as sick or healthy. C) ROC curves for the results of Tests 1, 2, and 3, plotting their sensitivities against their false positive rates.

On the other hand, test 3 (Figure 2B) adequately classifies sick individuals (high specificity), but is compromised by the possibility of false negatives. The ankle-brachial index is one example of a test with this behavior, since it is highly predictive of cardiovascular outcomes when results are abnormal, but normal results do not rule out this risk.^14^ Plotting the ROC curves for tests 1, 2, and 3 (Figure 2C) on the same graph demonstrates at which values sensitivity is optimized as a function of specificity.

In addition to analysis of points along the curve itself, ROC curves can also be used to indicate the dimension of an effect. The indicator most used is the area under the curve (AUC). The AUC is the result of integration of all of the points along the path of the curve and simultaneously computes sensitivity and specificity, offering an estimator of the overall behavior and accuracy of a test.^15^ The AUC offers an estimation of the probability of correctly classifying a random subject (test accuracy); an AUC of 0.7 indicates a 70% likelihood of correctly classifying the case. In general, AUC values are interpreted as follows: 0.5-0.6 (failed), 0.6-0.7 (worthless), 0.7-0.8 (poor), 0.8-0.9 (good), > 0.9 (excellent).^5^ There are various algorithms for calculating the AUC: if the ROC curve follows a smooth, curved, and symmetrical path (e.g. Figure 1B), a parametric estimator can be used, but if the curve has “steps” and asymmetries, then a non-parametric method must be chosen, which is more common in biomedical experiments.

The sensitivity and specificity points and the AUC estimate all have inferential properties with standard errors that are dependent on their effect sizes and the sample size. AUC statistics should therefore be presented together with their 95% confidence intervals, enabling comparison with the null hypothesis, for which AUC = 0.5.^15^

In Figure 2C, it can be observed that test 1 has the best overall performance, with an AUC of 0.96 (95% confidence interval [CI) 0.95-0.97; p < 0.01]. The AUC for test 2 is 0.77 (95%CI 0.75-0.79; p < 0.05) and the AUC for test 3 is 0.87 (95%CI 0.85-0.89; p < 0.05).

There are several circumstances in which a test may be chosen for its high sensitivity, even having low specificity (or vice-versa), if it is cheaper or more accessible, which is the case of rapid tests for HIV screening.^16^ In the examples, tests 2 and 3 achieve high sensitivity or specificity using specific cutoff values, even though overall performance is not superior to test 1.^5^ Researchers should therefore be careful not to generalize the AUC value as the only measure of test utility. In such situations a partial AUC value can even be calculated, within a set range of test values, maximizing comparability between different classification methodologies. However, such analyses are beyond the scope of this text.^17^^-^20

Construction of a ROC curve is not dependent on data following a normal distribution and is not substantially affected by sample asymmetry of positive or negative cases. However, it is fundamentally dependent on unequivocal *a priori* classification of cases and controls, generally using a gold standard diagnostic test or examination (e.g., autopsy or pathology).^5^^,^^21^^,^^22^ Sample size estimation for studies using ROC curves are primarily dependent on type I and II (power) errors and the estimated AUC for each test.^23^^,^^24^ Sample sizes and the most important characteristics of ROC curves can be estimated on-line using tools available on the easyROC website (http://www.biosoft.hacettepe.edu.tr/easyROC/).^23^^,^^25^

Use of ROC curves has been extended to evaluation of the performance of multivariate models for diagnosis, prognosis, machine learning (e.g., image or voice recognition), and data mining. Recently, Amato et al.^26^ conducted a cross-sectional study using clinical information and a bank of images from 110 patients who had undergone angiotomography of the aorta to predict identification of the artery of Adamkiewicz. Using multivariate analysis, they constructed a predictive model and evaluated its discriminatory properties using a ROC curve, showing that it enabled correct identification in 61% of the patients using a combination of nine covariates.

It is also possible to use ROC curves to represent ordinal classifications (e.g., mild, moderate, severe; stage I-IV; intensity from 0 to 4+), rather than binary classes,^27^ for two or more simultaneous classifications (ROC surface),^28^^-^30 and the results of ROC curves can be adjusted for other covariates using multivariate models (e.g., multiple logistic regression).^31^ However, these procedures demand input from an experienced statistician.

Finally, ROC curves are a very robust and intuitive option for description and comparison of classification models, in addition to providing support for choice of cutoff points to optimize categorization of phenomena. When employed in research, the parameters used must be precisely described in the methodology.

## References

[1] Forkmann T, Vehren T, Boecker M, Norra C, Wirtz M, Gauggel S (2009). Sensitivity and specificity of the Beck Depression Inventory in cardiologic inpatients: How useful is the conventional cut-off score?. J Psychosom Res.

[2] Rodríguez-Morán M, Guerrero-Romero F (2001). Fasting plasma glucose diagnostic criterion, proposed by the American Diabetes Association, has low sensitivity for diagnoses of diabetes in Mexican population. J Diabetes Complications.

[3] De Los Monteros AE, Parra A, Hidalgo R, Zambrana M (1999). The after breakfast 50-g, 1-hour glucose challenge test in urban Mexican pregnant women: its sensitivity and specificity evaluated by three diagnostic criteria for gestational diabetes mellitus. Acta Obstet Gynecol Scand.

[4] Hoo ZH, Candlish J, Teare D (2017). What is an ROC curve?. Emerg Med J.

[5] Metz CE (1978). Basic principles of ROC analysis. Semin Nucl Med.

[6] Corey D, Chang CK, Cembrowski GS (1984). Disheartened: need ROC curve. Am J Clin Pathol.

[7] Barraclough K (2012). Diagnosis: shifting the ROC curve. Br J Gen Pract.

[8] Sherwood EM, Bartels PH, Wied GL (1976). Feature selection in cell image analysis: use of the ROC curve. Acta Cytol.

[9] Kumar R, Indrayan A (2011). Receiver operating characteristic (ROC) curve for medical researchers. Indian Pediatr.

[10] Park SH, Goo JM, Jo CH (2004). Receiver operating characteristic (ROC) curve: practical review for radiologists. Korean J Radiol.

[11] Wei RJ, Li TY, Yang XC, Jia N, Yang XL, Song HB (2016). Serum levels of PSA, ALP, ICTP, and BSP in prostate cancer patients and the significance of ROC curve in the diagnosis of prostate cancer bone metastases. Genet Mol Res.

[12] Jahromi AS, Cina CS, Liu Y, Clase CM (2005). Sensitivity and specificity of color duplex ultrasound measurement in the estimation of internal carotid artery stenosis: A systematic review and meta-analysis. J Vasc Surg.

[13] Stein PD, Hull RD, Patel KC (2004). D-dimer for the exclusion of acute venous thrombosis and pulmonary embolism: a systematic review. Ann Intern Med.

[14] Doobay AV, Anand SS (2005). Sensitivity and specificity of the ankle-brachial index to predict future cardiovascular outcomes: a systematic review. Arterioscler Thromb Vasc Biol.

[15] Hanley JA (1989). Receiver operating characteristic (ROC) methodology: the state of the art. Crit Rev Diagn Imaging.

[16] Koblavi-Deme S, Maurice C, Yavo D (2001). Sensitivity and specificity of human immunodeficiency virus rapid serologic assays and testing algorithms in an antenatal clinic in Abidjan, Ivory Coast. J Clin Microbiol.

[17] Hsu MJ, Chang YC, Hsueh HM (2014). Biomarker selection for medical diagnosis using the partial area under the ROC curve. BMC Res Notes.

[18] Ma H, Bandos AI, Rockette HE, Gur D (2013). On use of partial area under the ROC curve for evaluation of diagnostic performance. Stat Med.

[19] Walter SD (2005). The partial area under the summary ROC curve. Stat Med.

[20] McClish DK (1989). Analyzing a portion of the ROC curve. Med Decis Making.

[21] Miot HA (2017). Assessing normality of data in clinical and experimental trials. J Vasc Bras.

[22] Miot HA (2016). Agreement analysis in clinical and experimental trials. J Vasc Bras.

[23] Kawada T (2012). Sample size in receiver-operating characteristic (ROC) curve analysis. Circ J.

[24] Hanley JA, McNeil BJ (1983). A method of comparing the areas under receiver operating characteristic curves derived from the same cases. Radiology.

[25] Goksuluk D, Korkmaz S, Zararsiz G, Karaagaoglu AE (2016). easyROC: An interactive web-tool for ROC curve analysis using R language environment. R J.

[26] Amato ACM, Parga JR, Stolf NAG (2018). Development of a clinical model to predict the likelihood of identification of the Adamkiewicz artery by angiotomography. J Vasc Bras.

[27] Miot HA (2020). Analysis of ordinal data in clinical and experimental studies. J Vasc Bras.

[28] Yang H, Carlin D (2000). ROC surface: A generalization of ROC curve analysis. J Biopharm Stat.

[29] Ramos PM, Gumieiro JH, Miot HA (2010). Association between ear creases and peripheral arterial disease. Clinics (São Paulo).

[30] Miot HA, Medeiros LMd, Siqueira CRS (2006). Association between coronary artery disease and the diagonal earlobe and preauricular creases in men. An Bras Dermatol.

[31] Schisterman EF, Faraggi D, Reiser B (2004). Adjusting the generalized ROC curve for covariates. Stat Med.

