# Supplementary material for: Use of ROC curves in clinical and experimental studies
Source: J Vasc Bras. 2020 Dec 11;19:e20200186. doi: 10.1590/1677-5449.200186 (PMC8218006; doi:10.1590/1677-5449.200186)
Supplement: Translation

# Translation: Aplicações da curva ROC em estudos clínicos e experimentais

**Authors:** Tatiana Cristina Figueira Polo, Hélio Amante Miot

**Affiliations:** 1 Universidade Estadual Paulista – UNESP, Faculdade de Medicina de Botucatu, Departamento de Dermatologia e Radioterapia, Botucatu, SP, Brasil.

Tanto as decisões da prática clínica como a operacionalização da pesquisa dependem de definições precisas e objetivas dos fenômenos ou dos desfechos (p. ex., doente vs. saudável, grave vs. leve, operável vs. inoperável). Entretanto, nem sempre essa classificação é direta ou inequívoca, podendo necessitar de elementos secundários para a categorização. Muitas condições, ainda, apresentam diferentes sistemas de diagnósticos, como *diabetes mellitus* (glicemia de jejum vs. teste oral de tolerância à glicose), isquemia crítica (parâmetro clínico vs. percentual de obstrução arterial) ou depressão (critérios DSM V vs. escala de Beck), o que repercute em diferentes sensibilidades e especificidades para cada classificação^1^^-^3.

Na verdade, na maior parte dos casos, os critérios utilizados para a classificação de desfechos não são completamente preditivos, levando à categorização incorreta de uma fração de doentes (falsos negativos) ou de indivíduos saudáveis (falsos positivos), tornando importante a comparação da eficiência dos sistemas classificatórios empregados.

Há uma série de estimadores estatísticos que são empregados na análise do desempenho de modelos classificatórios, e um dos mais utilizados é a curva ROC (*receiver operating characteristic*), que consiste em uma representação gráfica da performance de um modelo de dados quantitativos segundo sua taxa de sensibilidade (fração dos verdadeiros positivos) e a fração dos falsos positivos (1-especificidade), segundo diferentes valores do teste^4^^,^^5^. Sistemas classificatórios baseados em sintomas clínicos, escalas diagnósticas, achados radiológicos, dosagens de substâncias e, principalmente, escolha de pontos de corte para otimizar o desempenho de exames diagnósticos são as indicações mais usuais das curvas ROC^6^^-^11.

A Figura 1A exemplifica a representação de um exame de alta sensibilidade e alta especificidade para a classificação diagnóstica. Um teste hipotético (teste 1) apresenta duas curvas de distribuição de resultados para doentes e saudáveis. O ponto A1 representa o valor com melhor desempenho, considerado taxas de falsos positivos e falsos negativos (ponto de máxima entropia). O ponto A2 apresenta a maior especificidade, pois os valores superiores a ele não classificam nenhum falso negativo. Já o ponto A3 apresenta a maior sensibilidade, e os valores inferiores a ele não classificam nenhum falso positivo. Um exemplo de teste com essa performance é o dúplex de carótida, com alta preditividade de seus achados para estenose de carótida^12^.

**Figura 1:** A) Representação das curvas de distribuição de resultados para um teste hipotético que visa classificar pacientes como doentes ou saudáveis. B) Curva ROC dos resultados do teste 1 segundo sua sensibilidade e taxa de falsos positivos. O ponto A1 representa o valor do teste (ponto de corte) com maiores sensibilidade e especificidade (maior proximidade do canto superior esquerdo do gráfico). O ponto A2 representa o valor do teste a partir do qual se atinge a máxima sensibilidade (ausência de falsos positivos). O ponto A3 é o valor de máxima especificidade, abaixo do qual não deve haver falsos negativos.

A curva ROC do teste 1 (Figura 1B) possibilita acompanhar a variação da sensibilidade e da especificidade à medida que evoluem os pontos de corte, permitindo identificar facilmente os pontos A1, A2 e A3. À medida que se escolhe um ponto de corte com maior sensibilidade, necessariamente, se penitencia a classificação diagnóstica por sua menor especificidade e vice-versa.

Quanto mais a curva ROC se aproxima do canto superior esquerdo, melhor é a qualidade do teste quanto à capacidade para discriminar os grupos. Ainda, a linha de referência diagonal da curva ROC representa uma região de completa aleatoriedade do teste, incapaz de classificar tanto doentes como saudáveis (sensibilidade = especificidade).

É também possível comparar o desempenho de dois ou mais modelos classificatórios (ou exames diagnósticos), simultaneamente, a partir de suas curvas ROC. A Figura 2 acrescenta dois outros testes (testes 2 e 3) para classificar doentes e saudáveis. As curvas dos resultados do teste 2 (Figura 2A) mostram certa sobreposição entre os valores dos casos e controles, porém, apresentam um bom desempenho para valores baixos (alta sensibilidade). Um exemplo de exame com esse comportamento é a dosagem do dímero-D para o diagnóstico da trombose venosa profunda: valores muito baixos excluem o diagnóstico com segurança, enquanto valores altos exigem comprovação adicional (risco de falsos positivos)^13^.

**Figura 2:** A e B) Representação das curvas de distribuição de resultados para testes (testes 2 e 3) que visam classificar pacientes como doentes ou saudáveis. C) Curva ROC dos resultados dos Testes 1, 2 e 3 segundo as suas sensibilidades e taxas de falso-positivos.

O teste 3 (Figura 2B), por outro lado, classifica adequadamente os doentes (alta especificidade), mas é comprometido pela possibilidade de inclusão de falsos negativos. Um exemplo de exame com esse comportamento é o índice tornozelo-braquial, que apresenta grande preditividade de desfechos cardiovasculares quando seus resultados estão alterados, mas resultados normais não afastam o risco^14^. As curvas ROC dos testes 1, 2 e 3 (Figura 2C) permitem evidenciar, simultaneamente, os valores para os quais existe maior otimização da sensibilidade em função da especificidade.

Além da análise dos pontos da curva propriamente dita, é possível utilizar um indicador de dimensão do efeito para as curvas ROC. O mais empregado é a área sob a curva [*area under the curve* (AUC)]. A AUC é o resultado da integração de todos os pontos durante o trajeto da curva, e computa simultaneamente a sensibilidade e a especificidade, sendo um estimador do comportamento da acurácia global do teste^15^. A AUC fornece uma estimativa da probabilidade de classificação correta de um sujeito ao acaso (acurácia do teste); uma AUC de 0,7 reflete uma chance de classificação correta de 70% do caso. De forma geral, os valores da AUC são interpretados como: 0,5-0,6 (péssimo), 0,6-0,7 (ruim), 0,7-0,8 (pobre), 0,8-0,9 (bom), > 0,9 (excelente)^5^. Há diferentes algoritmos para calcular a AUC: quando a curva ROC tem trajeto curvilíneo simétrico e suave (p.ex., Figura 1B), pode-se empregar a estimativa paramétrica; por outro lado, quando a curva apresenta “degraus” e assimetrias, deve-se optar pelo método não-paramétrico, que é mais usual em experimentos biomédicos.

Tanto os pontos de sensibilidade e especificidade como a estimativa da AUC possuem propriedades inferenciais cujos erros-padrão dependem das suas dimensões do efeito e do tamanho da amostra. As estatísticas da AUC devem ser referidas, portanto, segundo seu intervalo de confiança de 95%, permitindo sua comparação com a hipótese nula, de AUC = 0,5^15^.

Na Figura 2C, pode-se observar que o teste 1 apresenta melhor desempenho global, e a sua AUC é de 0,96 [intervalo de confiança (IC) 95% 0,95-0,97; p < 0,01]. Já para o teste 2, a AUC é de 0,77 (IC 95% 0,75-0,79; p < 0,05); e, no teste 3, a AUC é de 0,87 (IC 95% 0,85-0,89; p < 0,05).

Há diversas circunstâncias em que um teste pode ser optado por sua alta sensibilidade, mesmo com menor especificidade (ou vice-versa), desde que sejam mais baratos ou disponíveis, como ocorre com os testes rápidos para triagem do HIV^16^. No caso dos testes 2 e 3, eles atingem alta sensibilidade ou especificidade a partir de certos valores de corte, apesar de não apresentarem comportamento global superior ao teste 1^5^. Por esse motivo, o pesquisador deve estar atento para não generalizar unicamente o valor de AUC como a utilidade do teste. Nessas situações, é possível, inclusive, calcular o valor parcial da AUC, dentro de uma faixa de valores do teste, o que maximiza a comparação de diferentes metodologias classificatórias. Entretanto, essa análise ultrapassa o escopo deste texto^17^^-^20.

A construção de uma curva ROC não depende de que os dados apresentem distribuição normal e não é substancialmente afetada pela assimetria amostral de casos positivos ou negativos. Entretanto, depende fundamentalmente da inequívoca classificação *a priori* de casos e controles, geralmente a partir de um exame padrão-ouro para o diagnóstico (p.ex., autópsia ou anatomopatologia)^5^^,^^21^^,^^22^. A estimativa do tamanho de amostra de estudos que utilizem curvas ROC depende principalmente dos erros tipo I, II (poder) e da AUC estimada para cada teste^23^^,^^24^. O tamanho amostral e as principais características de curvas ROC podem ser estimados on-line a partir do site easyROC (http://www.biosoft.hacettepe.edu.tr/easyROC/)^23^^,^^25^.

O emprego das curvas ROC foi estendido para a avaliação do desempenho de modelos multivariados para diagnósticos, prognósticos, aprendizado de máquinas (p.ex., reconhecimento de imagens ou de voz) e mineração de dados. Recentemente, Amato et al.^26^ conduziram um estudo transversal que utilizou informações clínicas e um banco de imagens de 110 pacientes submetidos a angiotomografias de aorta para prever a identificação da artéria de Adamkiewicz. A partir da análise multivariada, foi criado um modelo de predição cuja discriminação foi avaliada pela curva ROC, permitindo a correta identificação em 61% dos pacientes a partir da combinação de nove covariáveis.

É possível ainda se utilizar curvas ROC para representar classificações ordinais (p.ex., leve, moderado e grave; estágio I-IV; intensidade 0 a 4+) ao invés de binárias^27^, para duas ou mais classificações simultâneas (*ROC surface*)^28^^-^30, assim como se pode ajustar o resultado de curvas ROC para outras covariáveis a partir de modelos multivariados (p.ex., regressão logística múltipla)^31^. Esses procedimentos, no entanto, demandam a assistência de um estatístico experiente.

Finalmente, as curvas ROC são alternativas bastante robustas e intuitivas para a descrição e comparação de modelos classificatórios, além de subsidiar a escolha de pontos de corte para otimizar a categorização de um fenômeno. Seu emprego na pesquisa exige descrição precisa na metodologia quanto aos parâmetros de uso.
